# Editorial to irrigated contact‐force sensing catheter may be beneficial for redo ablation of slow–fast atrioventricular nodal reentrant tachycardia in pediatric and adolescent patients

**DOI:** 10.1002/joa3.13015

**Published:** 2024-03-15

**Authors:** Pi‐Chang Lee

**Affiliations:** ^1^ Department of Pediatrics Taichung Veterans General Hospital Taichung City Taiwan

**Keywords:** ablation, atrioventricular nodal reentrant tachycardia, children, irrigated contact‐force sensing catheter

Atrioventricular nodal reentrant tachycardia (AVNRT) is a common arrhythmia in pediatric and adolescent patients with regular supraventricular tachycardia. Selective radiofrequency (RF) catheter ablation or modification of the slow pathway has afforded an ideal method to treat most patients with AVNRT. Recently, cryoablation has been widely used to achieve a good result and prevent complete AV block, especially in young patients. However, successful arrhythmia elimination may be not achieved by conventional ablation catheters in certain cases. The use of irrigated contact‐force sensing (ICFS) catheters has been increasing over time for RF catheter ablation of several arrhythmic substrates in both adults and children.^1^ The ICFS catheters can give a greater power because they are not limited by temperature and impedance rise at the catheter–tissue interface, gaining larger and deeper lesions in power‐controlled mode, as opposed to nonirrigated ablation catheters, which are inhibited by the temperature increase for the more effective lesions creation. Previous literature suggested to use the lowest programmable irrigation flow rate to obtain the benefits of irrigated catheters for AVNRT ablation, such as delivering higher power and creating more durable and effective lesions on the slow pathway, without extending the lesion size excessively, minimizing the risks of damage on the AV node.

The compact AV node is located inside the triangle of Koch (bounded by the tendon of Todaro, septal leaflet of the tricuspid valve, and coronary sinus ostium), and its size is around 5–7 mm in length and 2–5 mm in width. In addition, the His bundle is located at the summit of Koch's triangle, where the septal leaflet interconnects the tendon of Todaro. Besides, the compact AV node is a complex histological structure, which consists of a loose transitional zone of cells blending and extending into the surrounding atrial myocardium.^2^ These transitional cells are found in Koch's triangle and merge with the compact node. However, the contour of Koch's triangle may be small or even horizontal in some patients, which will cause a higher risk of heart block during RF energy application. Distal to the node is the bundle of His and bundle branches, which are tracts of specialized cells (smaller than atrial myocardial cells), encased by insulating sheaths of fibrous tissue (central fibrous body).^3^ The electrogram characteristics inside Koch's triangle around the compact AV node display a relatively lower range of voltages exported and analyzed from the noncontact mapping system.^4^ The Koch's triangle did reveal a lower range of voltages compared with outside Koch's triangle during sinus rhythm and atrial pacing, possibly due to the transitional cell distribution including multiple layers associated with a marked variability of fiber orientation produced by longitudinal and circumferential fibers and anisotropic conduction.

The RF energy will generate heat‐induced tissue necrosis to manage the arrhythmia substrates. The RF energy application will be unsuccessful if inadequate temperature for tissue heating. However, the target temperatures greater than 50°C may not provide additional benefit, and the low temperature required for successful slow pathway ablation may help to limit lesion size and the occurrence of complete AV block. Therefore, a considerate power titration with automatic power adjustment to achieve the predetermined target temperature is useful to avoid such a preventable complication. The presence of junctional rhythm has been considered to be a sensitive marker of successful slow pathway ablation. However, in rare cases, junctional rhythm was absent despite multiple RF applications delivered over a large area in the Koch's triangle, and successful ablation was achieved in the absence of a junctional rhythm.^5^ The success rate of slow pathway ablation is high, but transient or complete heart block requiring permanent pacing still occurs occasionally. The incidence has been reported to be higher in those patients with abnormal anatomy of AV node, especially if there is a posterior displacement of the fast pathway or compact AV node.^4^ Different energy applications were developed to avoid such a life‐threatening complication. Unlike RF energy ablation, cryoablation can create a lesion that is initially reversible and may prevent the complication of a complete heart block. Although transient PR prolongation and 2:1 AV block were occasionally noted, complete recovery of AV nodal conduction resumed upon rewarming possibly due to the AV node particularly resistant to cryothermal injury. Therefore, accelerated junctional tachycardia will not be observed during cryoablation. In addition, given catheter adherence to underlying endocardium, testing for slow pathway conduction during the cryoablation can be also performed simultaneously. The termination of AVNRT during cryoablation is also a good endpoint for successful ablation. Cryoablation seems to be associated with a similar acute success rate and recurrence rate compared with RF ablation in both adult and pediatric patients with AVNRT. For RF energy ablation, the persistent dual AV nodal physiology with or without echo beats was not associated with higher recurrence rates than the complete elimination of dual AV nodal physiology, if AVNRT remained noninducible on and off isoproterenol.

Nonirrigated catheters are conventionally used for AVNRT ablation in a temperature‐controlled mode since deep lesions are not usually required for successful slow pathway ablation. Nevertheless, nonirrigated catheters may not generate adequate lesions for effective slow pathway modification in certain cases, causing persistent tachycardia inducibility or recurrences despite induction of slow junctional rhythm during ablation. Thus, the ICFS catheters may be an alternative option in these cases. Besides, the real‐time monitoring of the catheter‐endocardial contact can be useful to get even more effective lesions, providing earlier discontinuation of ineffective RF applications in case of inadequate contact force (CF), consequently reducing the total number of RF pulses and total RF time. Simultaneously, CF monitoring allows to avoid dangerous high CF values and high impedance drops, reducing the risk of perforation and vascular injury on coronary arteries located in the posteroseptal region, such as the AV nodal artery. In the present study, the authors observed no case of iatrogenic AV block nor coronary artery injury, maintaining low CF values with low irrigation flow rate and precisely targeting the slow pathway by electroanatomic mapping. Because the major safety concern the ICFS catheters for AVNRT ablation is the greater risk of AV block than the adult population, the ICFS catheter is not appropriate for younger children with very small body sizes and short distances between slow and fast pathways. To date, cryoablation or nonirrigated RF ablation still represents the safest approach for these patients.

## CONFLICT OF INTEREST STATEMENT

Author declare no conflict of interests for this article.

## References

[1] Ciriello GD , Correra A , Russo MG , Sarubbi B . Irrigated contact force sensing catheter for redo ablation of slow–fast atrioventricular nodal reentrant tachycardia in pediatric and adolescent patients: a case series. J Arrhythm. 2023;00:1–4. 10.1002/joa3.12967 PMC10848577 38333387

[2] Lee PC , Chen SA , Hwang B . Atrioventricular node anatomy and physiology: implications for ablation of atrioventricular nodal reentrant tachycardia. Curr Opin Cardiol. 2009;24(2):105–112. 19225293 10.1097/HCO.0b013e328323d83f

[3] Anderson RH , Ho SY . The architecture of the sinus node, the atrioventricular conduction axis, and the internodal atrial myocardium. J Cardiovasc Electrophysiol. 1998;9(11):1233–1248. 9835269 10.1111/j.1540-8167.1998.tb00097.x

[4] Lee PC , Tai CT , Lin YJ , Liu TY , Huang BH , Higa S , et al. Noncontact three‐dimensional mapping guides catheter ablation of difficult atrioventricular nodal reentrant tachycardia. Int J Cardiol. 2007;118(2):154–163. 17023073 10.1016/j.ijcard.2006.08.003

[5] Hsieh MH , Chen SA , Tai CT , Yu WC , Chen YJ , Chang MS . Absence of junctional rhythm during successful slow‐pathway ablation in patients with atrioventricular nodal reentrant tachycardia. Circulation. 1998;98(21):2296–2300. 9826317 10.1161/01.cir.98.21.2296

